# National survey of training of psychiatrists on advance directives to refuse treatment in bipolar disorder

**DOI:** 10.1192/pb.bp.116.055343

**Published:** 2017-12

**Authors:** Richard Morriss, Mohan Mudigonda, Peter Bartlett, Arun Chopra, Steven Jones

**Affiliations:** 1 University of Nottingham; 2 Royal Edinburgh Hospital; 3 University of Lancaster

## Abstract

**Aims and method** To determine features associated with better perceived quality of training for psychiatrists on advance decision-making in the Mental Capacity Act 2005 (MCA), and whether the quality or amount of training were associated with positive attitudes or use of advance decisions to refuse treatment (ADRTs) by psychiatrists in people with bipolar disorder. An anonymised national survey of 650 trainee and consultant psychiatrists in England and Wales was performed.

**Results** Good or better quality of training was associated with use of case summaries, role-play, ADRTs, assessment of mental capacity and its fluctuation. Good or better quality and two or more sessions of MCA training were associated with more positive attitudes and reported use of ADRTs, although many psychiatrists would never discuss them clinically with people with bipolar disorder.

**Clinical implications** Consistent delivery of better-quality training is required for all psychiatrists to increase use of ADRTs in people with bipolar disorder.

The Mental Capacity Act 2005 (MCA) in England and Wales provides a legal framework for personal welfare and financial decisions to be made in advance by individuals, who later due to an impairment or disturbance of functioning in the mind or brain, may be unable to make these decisions for themselves. If capacity is not present, a decision can be made on behalf of the person based on what is in their best interests taking consideration of their wishes using three specific provisions of the MCA for advance decision-making:

1. advance decision to refuse treatment (ADRT), a legally binding provision preventing specific treatment;
2. advance statement of wishes and feelings, a non-legally binding statement of preferences for treatment, and/or personal and financial affairs;
3. lasting power of attorney, a legally binding direction identifying who will look after the person's personal and financial affairs.

Clinicians and their employers, especially psychiatrists, are legally required to ‘have regard to’ MCA guidance and, if later asked, prove that they did.^1^ Therefore there is an expectation that psychiatrists receive training in the MCA However, the methods and amount of training that psychiatrists should receive are not specified, nor has the quality or amount of training been related to attitudes or use of ADRTs in practice. We chose to examine the attitudes to and use of ADRTs by psychiatrists as these may be seen as restrictive in terms of treatment offered by psychiatrists to people with bipolar disorder. In a national survey of general adult and old age psychiatrists in England and Wales, we wished to explore how the quality and amount of training they have received may be associated with their implementation of ADRTs in people where capacity is lost (e.g. mania, severe depression) and regained (e.g. bipolar disorder) to complement a survey of patient experience in bipolar disorder.^2^

## Method

### Objectives and design

Our objectives were:

1. to determine what aspects of training in the MCA were associated with higher or lower perceived quality of training in the view of psychiatrists; and
2. to examine whether the quality and amount of training were associated with reported attitudes or use of ADRT in people with bipolar disorder.

We anticipated that high-quality training may be required to overcome professional resistance to the use of ADRTs should any be present.

### Participants

Inclusion/exclusion criteria were:

- participants practice within England and Wales, i.e. the jurisdiction of the MCA
- specialise in either general adult or old age psychiatry
- they were consultant psychiatrists or in training grades (CT1-CT3, ST4-ST6).

### Procedure

We aimed to recruit a national sample of 500 psychiatrists in a 12-month period for the survey. No data were available for a formal power calculation. The study was advertised with the help of the National Institute of Health Research-funded Mental Health Research Network (MHRN) and the Royal College of Psychiatrists. The College agreed to publicise the study by tweeting the link to the survey and the study team also attended a national conference organised by the College to publicise the study. Consultants, senior and junior trainees in general adult and old age psychiatry were selected from different regions to ensure maximum variance of practical clinical experience. To maximise the participation rate of psychiatrists and the frankness of their responses, we anonymised the survey, not asking for personal information such as age, gender or workplace, and placed it online or if they preferred we administered it face to face, by telephone or posted it.

### Measures

The survey was divided into nine sections that addressed the following topics:

- Section A: Preliminary information – position, years since qualification, place of work (e.g. in-patient, crisis team), geographic location.
- Section B: MCA training – how many sessions attended, whether mandatory, how recent, whether training considered advance decision-making that included ADRTs, nature of training, quality of training (e.g. in your opinion how much of the training focused on advance decision-making (including ADRTs) – a significant amount, a reasonable amount, a minimal amount, none?).
- Section C: ADRTs and bipolar disorder – whether psychiatrists had experience of patients making ADRTs, whether they had advised on making ADRTs, content of ADRTs, factors influencing their decision to advise regarding ADRTs.
- Section D: ADRTs and other conditions – content of ADRTs.
- Section E: ADRTs and the Mental Health Act 1983 – whether psychiatrists had encountered ADRTs in context of patients admitted to psychiatric units or sectioned under the Mental Health Act.
- Section F: ADRTs in clinical practice – how often should they be discussed (e.g. in your opinion how often do you feel that discussion of ADRTs should take place – at every consultation, every 6 months, at care programme approach meetings, only when I think I might be relevant, only when another health or social care professional raises the topic, only if the patient or carer raises the topic or never?).
- Section G: Advance statement of wishes and feelings – whether psychiatrists had experience of patients using these; what was contained, whether the frequency was changing among people with bipolar disorder.
- Section H: ADRTs and implementation of the MCA – whether psychiatrists had experience of patients using ADRTs and their contents.
- Section I: Lasting power of attorney – whether psychiatrists had experience of patients making lasting powers of attorney, who advised on these.

### Analysis

Descriptive statistics were employed in the survey to explore the professional characteristics of psychiatrists and their experience of training. Univariate analysis indicated that several demographic or service provision factors may be associated with the use of the MCA. Binary logistic regression was applied to three separate analyses:

1. the quality of training (dependent variable) perceived by psychiatrists was explored in relation to the methods, site and content of training
2. the quality of training (dependent variable) was then related to attitudes and experiences of psychiatrists to implementing ADRTs in their clinical practice
3. the amount of training (dependent variable) was related to their attitudes and experiences of implementing ADRTs.

Checks for collinearity were applied by exploring the Spearman correlations between the independent variables that might enter the logistic regression. None of the independent variables were excluded because of collinearity. Odds ratios (ORs) and 95% confidence intervals (CIs) are presented for any significant variables.

## Results

A total of 650 psychiatrists were recruited for the survey. Table 1 shows the grade, work setting, country of medical training and duration of time since medical qualification of this sample. Within the sample, there were 374 (57.5%) consultants in general adult or old age psychiatry, and the remainder were trainees, with a slight majority qualified in medicine outside the UK. Psychiatrists were recruited for the study between May 2011 and June 2012. Of 607 respondents who identified the geographic location of their work, 133 (21.9%) were from the West Midlands, 116 (19.1%) from the East Midlands, 80 (13.2%) from the South West, 116 (19.1%) from the South East, 74 (12.2%) from the East of England, 46 (7.6%) from London and 10 (1.6%) from the North West of England.

**Table 1:** Professional characteristics and nature of Mental Capacity Act 2005 training of psychiatrists (*n* = 650)

Work characteristic	*n*	%
Grade		
Consultant general adult psychiatry	283	43.5
Consultant old age psychiatry	91	14.0
ST4–6 trainee	111	17.1
CT1–3 trainee	130	20.0
Missing	35	5.4

Main work setting		
Community mental health team	349	53.7
In-patient	216	33.3
Crisis team/EIP/ACT	77	11.9
Missing	8	1.2

Years since medical qualification		
0–10	210	32.3
11–20	241	37.1
21–30	146	22.5
30+	51	7.8
Missing	2	0.3

Country of medical qualification		
UK	306	47.1
European Union	51	7.8
Outside European Union	288	44.3
Missing	5	0.8

Number of training sessions		
0	55	8.5
1	128	19.7
2	183	28.2
3	113	17.4
>3	169	26.0
Trained but missing data	2	0.3

Method of training^a^		
Case examples	491	75.5
Role-play	82	12.6
Watch video	44	6.8
None of these	86	13.2

Source of training^a^		
Local NHS trust	489	75.2
Royal College of Psychiatrists	133	20.5
Legal or solicitor	48	7.4
Pharmaceutical company	35	5.4
Other	89	13.7

Perceived quality of training		
Excellent	24	4.0
Very good	153	25.7
Good	269	45.2
Average	134	22.5
Below average	12	2.0
Missing	58	8.9

Primary reason for attending		
Mandatory NHS trust training	172	28.9
Approved clinician training	194	32.6
Educational event	128	71.5
Personal interest	79	13.3
Other	22	3.7
Missing	55	8.4

ACT, assertive community treatment; EIP, early intervention in psychosis; NHS, National Health Service.

a. Categories are not mutually exclusive.

Table 1 shows the number of training sessions, methods used for training, source of the training, quality of training and reasons for attending the training: 595 (91.5%) had attended at least one training session on the MCA; 465 (71.5%) had attended two or more sessions; and 326 (50.1%) had been to a training session in the previous year. Of the 595 psychiatrists trained in the MCA 489 (75.2%) had been trained by their local National Health Service (NHS) trust. The quality of the training was perceived to be high, with 446 (75.0% receiving training) rating it as good, very good or excellent (Table 1). However, 209 (35.1% receiving training) psychiatrists stated that either minimal or no attention was paid to ADRTs in the training sessions.

Table 2 examines the binary multiple logistic regression associations between the quality of training and the methods of training, the site of training, the number of training sessions and topics covered in the training. Compared with average or poor training, good or better (very good or excellent) training was associated positively with the use of case summaries, role play, coverage of advance decision-making (including ADRTs) and assessment of capacity. Video feedback was only carried out in good or better quality of training (44 or 9.9%, Fisher's exact 2-tailed test *P* < 0.001). Average or poor training was associated with training in their own NHS trust compared with good or better training (Table 2). In relation to the specific use of advance decision-making including ADRTs and the need to be able to assess fluctuating capacity in conditions such as bipolar disorder with highly variable severity and therefore capacity, it is notable that even good or better-quality training covered these issues in only just over 45% and 37% of cases respectively.

**Table 2:** Content and method of training related to perceived quality of training in the Mental Capacity Act 2005^a^ (*n*=588)

	Quality of training						
	Good or better (*n* = 444)		Average or worse (*n* = 144)		Multivariate statistics		
Training characteristic	*n*	%	*n*	%	Odds ratio	95% CI	*P*
Used role-play	76	17.1	26 6	4.1	3.32	1.37–8.07	0.008

Training in advance decision-making^b^	203	45.6	26	17.8	2.58	1.54–4.31	<0.001

Capacity assessment	410	92.3	107	74.3	2.80	1.56–5.02	0.001

Training in their NHS trust	355	80.0	132	91.7	0.39	0.20–0.77	0.007

NHS, National Health Service.

a. 55 psychiatrists received no Mental Capacity Act training, 7 missing responses.

b. Including advance decision to refuse treatment.

Only 94 (14.5%) of surveyed psychiatrists had encountered a patient with bipolar disorder who had made an ADRT; 136 (20.9%) had encountered a patient with bipolar disorder who had made an oral or written statement of wishes and feelings; and 91 (14.0%) had encountered a patient with bipolar disorder who had made a lasting power of attorney relating to health or personal welfare. Of the 259 psychiatrists expressing an opinion, 208 (80.3%) considered that the number of people with bipolar disorder making ADRTs had remained the same since the implementation of the MCA in 2007, and 41 (15.8%) considered that it had increased by less than 10%. Of the 252 psychiatrists expressing a view regarding statements of wishes and feelings by people with bipolar disorder, 187 (74.2%) thought that the frequency remained the same since the MCA came into force, and 46 (18.3%) that it had increased by less than 10%.

Table 3 displays the binary multiple logistic regression associations between the quality of training and the discussion of ADRT with patients with bipolar disorder or other patients who may lose mental capacity but then regain it. Compared with average or poor training, good or better training was associated with fewer psychiatrists who never discuss ADRTs with patients, and fewer psychiatrists who believed that they had insufficient time to discuss ADRTs with patients. Table 4 shows that compared with only receiving one training session on the MCA receiving two or more training sessions was associated with more psychiatrists discussing ADRTs at care programme approach meetings and fewer psychiatrists who believed that they had insufficient training to discuss ADRTs with patients. There were no other associations between the quality of MCA training or number of MCA training sessions and reported practice or beliefs about implementing ADRTs.

**Table 3:** Relationship between quality of training in the Mental Capacity Act 2005 and barriers to implementing ADRTs^a^

	Quality of training						
	Good or better (*n* = 444)		Average or worse (*n* = 144)		Multivariate statistics		
Training characteristic	*n*	%	*n*	%	Odds ratio	95% CI	*P*
Never discuss ADRTs	96	21.5	48	32.9	0.53	0.35–0.79	0.010

Insufficient time to do ADRTs	177	39.7	79	54.1	0.57	0.37–0.88	0.002

ADRTs, advance decisions to refuse treatment.

a. 55 psychiatrists received no Mental Capacity Act training, 7 missing responses on quality of training and 3 missing responses on amount of training.

**Table 4:** Relationship between amount of training in the Mental Capacity Act 2005 and barriers to implementing ADRTs^a^

	Amount of training				Multivariate statistics		
Training characteristic	⩾ 2 sessions (*n* = 465)		1 session (*n* = 127)		Odds ratio	95% CI	*P*
Discuss ADRTs routinely at care programme approach meetings	77	16.6	11	8.7	2.372	1.17–4.83	0.017

Insufficient training to do ADRTs	178	38.3	80	63.8	0.41	0.27–0.63	<0.001

ADRTs, advance decisions to refuse treatment.

a. 55 psychiatrists received no Mental Capacity Act training, 7 missing responses on quality of training and 3 missing responses on amount of training.

However, 206 (46.3%) psychiatrists would not discuss ADRTs even if the person with bipolar disorder or carer raised it, and even after good or better training 96 (21.5%) would never discuss ADRTs. Furthermore, 177 ( 39.7%) and 178 (38.3%) of psychiatrists still believed they had insufficient training and time to discuss ADRTs in clinical practice despite good or better training and two or more training sessions respectively.

## Discussion

Although the need for training of psychiatrists and other clinical health staff in the MCA is often recommended or even required,^1,3,4^ and clinical guidelines also support the importance of considering the MCA in people with bipolar disorder,^5^ there is an assumption that all training is likely to help clinicians become more familiar with the MCA and that such training will improve attitudes and use in practice of the MCA by psychiatrists. We found that there was plenty of training in the MCA being offered to and taken up by psychiatrists at trainee and consultant level; 92% of trainee and consultant psychiatrists had received at least one training session on the MCA, with 50% receiving the training in the past year. Although 75% of psychiatrists rated their training in the MCA as good or better, ADRTs were only covered in 65% of the MCA training.

Psychiatrists preferred MCA training that was not didactic and merely information giving, rating training as good or better that utilised discussion of the MCA in relation to case summaries, used role-play, and covered topics such as ADRT, the assessment of capacity and the assessment of fluctuating capacity. Although the assessment of mental capacity was usually covered in MCA training, the topic of fluctuating capacity was rarely discussed, whereas the potentially challenging issue of ADRTs was discussed in only 39% of MCA training attended by psychiatrists. Therefore in the view of the authors, training of psychiatrists was rarely of sufficient quality to meet the needs of people with bipolar disorder under the MCA Training arranged by NHS trust was not perceived to be as good as training provided by the Royal College of Psychiatrists, law firms or other external agencies. The reasons for this view are unclear.

There was some evidence that good- or better-quality MCA training received by psychiatrists was associated with fewer psychiatrists reporting that they would never discuss ADRTs under any circumstances. Receipt of two or more sessions of MCA training was associated with an increased likelihood that ADRTs would be discussed routinely in multidisciplinary care programme approach meetings. Both better quality and more training sessions were associated with a reduced likelihood that psychiatrists had insufficient time to address ADRTs. Although these data are associations and not a comparison of interventions delivered in a randomised controlled trial, there was some evidence that higher-quality training and more than one training session may be helpful in both improving the attitudes to and use in clinical practice of ADRTs by psychiatrists in patients with bipolar disorder or other patients who lose and then regain mental capacity. Another alternative explanation is that psychiatrists who are interested in helping people with bipolar disorder through the MCA attend more than one session of training and find better-quality training.

Nevertheless offering training in the MCA that psychiatrists perceive as good or better quality seems insufficient to improving their attitudes to ADRTs and their use in practice in people with bipolar disorder. Even after good or better training, 22% of psychiatrists would never discuss ADRTs under any circumstances, 46% would not discuss ADRTs even if the person with bipolar disorder or carer raised it, and 39% believed they had insufficient training and time to discuss ADRTs in clinical practice. These findings chime with the experience of people with bipolar disorder in a national survey we carried out^2^ where neither knowledge nor use of ADRTs were associated with seeing a psychiatrist, although knowledge and use of ADRTs were associated with seeing other mental health professionals and attendance at peer support groups.

A strength of the survey was that to our knowledge it is the first of its sort inquiring into quality of training of psychiatrists and relating it to their attitudes and use of ADRTs with people with bipolar disorder. The survey was large, national and deliberately anonymised so that psychiatrists would feel able to comment frankly without any possible constraint. We judged that this advantage of the methodology outweighed the disadvantage that we do not know how many psychiatrists had the opportunity to take part in the survey but decided not to. We also do not know much about the characteristics of psychiatrists in terms of the demographic characteristics of who did or did not take part in the survey. A further limitation was that this survey was completed 4 years ago so the quality of training and use of ADRTs in clinical practice may have improved. Furthermore, by concentrating on MCA training in relation to ADRTs in bipolar disorder, we cannot comment on other aspects of MCA training on other forms of advance decision-making, application of ADRTs in people who are less likely to regain mental capacity and deprivation of liberty.

The findings confirm those of a 4-year re-audit study where increases in MCA training and improved documentation had a minimal impact on the recording of the MCA by psychiatrists in patient records.^6^ There seems to be some consistency in studies of advance planning that the therapeutic relationship between mental health professionals, including psychiatrists, and their patients is improved with advance planning.^7,8^ The House of Lords heard much evidence that the implementation of the MCA had failed to make much of an impact on clinical practice in the way that was intended, and made 39 recommendations to improve the implementation of the MCA^3^ We have not had the opportunity to study the effects of these recommendations but note that none of these relate to the quality or amount of training that psychiatrists or other health professionals receive in relation to the MCA. The Academy of Royal Medical Colleges were asked to report on measures to improve the uptake of the MCA^3,4^ So far it has organised educational events on the MCA but has not made recommendations on the content, form or amount or frequency of training that psychiatrists or other health professionals should receive in relation to the MCA.^9^

Therefore we conclude that there is a need to improve the quality of training that psychiatrists receive on the MCA so that fluctuating capacity and ADRTs are covered, and that techniques such as case summaries and role-play are employed to improve confidence and competencies of psychiatrists in its use. There may be a case for adding training in the MCA to mandatory training under the Mental Health Act section 22 training regulations. There is a need for further implementation research on ways to improve the knowledge and use of the MCA including ADRTs, by people with bipolar disorder or other conditions where capacity is lost and then regained, and also on how to improve the attitudes of psychiatrists and assist them further to discuss ADRTs with people who have bipolar disorder or similar conditions.
